# Hypohidrotic Ectodermal Dysplasia: Prosthetic and Endodontic Management

**DOI:** 10.5005/jp-journals-10005-1056

**Published:** 2010-04-15

**Authors:** Deshraj Jain, Sandhya Jain, Alok Kumar, Tripty Rahangdale

**Affiliations:** 1 Professor and Head, Department of Prosthodontics, Government College of Dentistry, Indore, Madhya Pradesh, India; 2 Professor and Head, Department of Orthodontics, Government College of Dentistry, Indore, Madhya Pradesh, India; 3 Postgraduate Student, Department of Prosthodontics, Government College of Dentistry, Indore, Madhya Pradesh, India; 4 Postgraduate Student, Department of Prosthodontics, Government College of Dentistry, Indore, Madhya Pradesh, India

**Keywords:** Ectodermal dysplasia, anodontia, overdentures, complete dentures.

## Abstract

Individuals affected by ectodermal dysplasia syndromes have abnormalities of the glands, tooth buds, hair follicles, and nail development. Oral finding in ectodermal dysplasia patient are significant and can include multiple abnormalities of the dentition such as anodontia, hy-podontia or malformed and widely spaced peg like teeth, loss of occlusal vertical dimension, protuberant lips and lack of normal alveolar ridge development. This clinical report describes a combined surgical, pedodontic, and prosthodontic approach for the treatment of a patient with hypohidrotic ectodermal dysplasia.

## INTRODUCTION

Ectodermal dysplasia (OMIM &305100)^1^ is a heterogeneous group of disorders characterized by developmental dystrophies of ectodermal structures, such as hypohidrosis, hypotrichosis, onchodysplasia and hypodontia or anodontia.

Synonyms^2^ of ectodermal dysplasia are anhydrotic ectodermal dysplasia, ectodermal dysplasia anhidrotic and Christ-Siemens-Touraine syndrome.

Individuals affected by ectodermal dysplasia syndromes have abnormalities of the glands, tooth buds, hair follicles, and nail development. Patient with ED having prominent supraorbital ridge, saddle nose, frontal bossing. The nose may appear pinched and alae nasi is hypoplastic. The skin of patient may appear hypo pigmented or maculopapular eruptions during infancy.^3^

Some ectodermal dysplasia syndromes are mild, while others are devastating. Other symptoms include deficient tears and saliva, poorly functioning mucous membranes, frequent respiratory infections, hearing or vision deficits, missing fingers or toes, cleft lip or palate, problems with the immune system, sensitivity to light, lack of breast development, and other abnormalities of the ectoderm.

Oral finding in ectodermal dysplasia patient are significant and can include multiple abnormalities of the dentition such as anodontia, hypodontia or malformed and widely spaced peg like teeth, loss of occlusal vertical dimension,^4^ protuberant lips and lack of normal alveolar ridge development.

With little or no dental support,^5^ a hypoplastic maxilla and mandible result in bite collapse and narrowing of alveolar ridge.

ED is inherited as an X-linked recessive trait, and has two major types:

1. Hypohidrotic or anhidrotic (Christ-Siemens-Touraine syndrome)
2. Hidrotic EDs (Clouston syndrome).^6^

The most common condition among the EDs is hypohi-drotic ectodermal dysplasia (HED). This is the more severe form and is associated with^7^ hypodontia or anodontia, hypotrichosis (fine, sparse blond hair, including a decreased density in both eyebrows and eyelashes), and hypohidrosis or anhidrosis.

This X-linked recessive disorder affects males and is inherited through female carriers. The diagnostic tool is the typical clinical physiognomy.^2^ A distinct form of X-linked hypohidrotic ectodermal dysplasia is associated with immune deficiency.^1^

Early and extensive dental treatment is needed throughout childhood because of the absence of most of the deciduous and permanent dentition. ED is usually a difficult condition to manage prosthodontically, because of the typical oral deficiencies and afflicted individuals are quite young^8^ to receive extensive prosthodontic treatment, which restores their appearance, for the development of positive self-image.

This case report essentially emphasizes on a different approach of prosthetic management of appearance, functionality of treatment in the form of denture provided. A multidisciplinary team approach for the treatment is recommended in these cases. This clinical report describes a combined surgical, pedodontic, and prosthodontic approach for the treatment of a patient with hypohidrotic ectodermal dysplasia.

## CLINICAL REPORT

A 13-year-old boy had reported to the Department of Prosth-odontics, College of Dentistry, Indore, India with a chief complaint of missing teeth and difficulty in chewing. The parents noticed presence of only few teeth in his mouth.

**Figs 1A and B:**
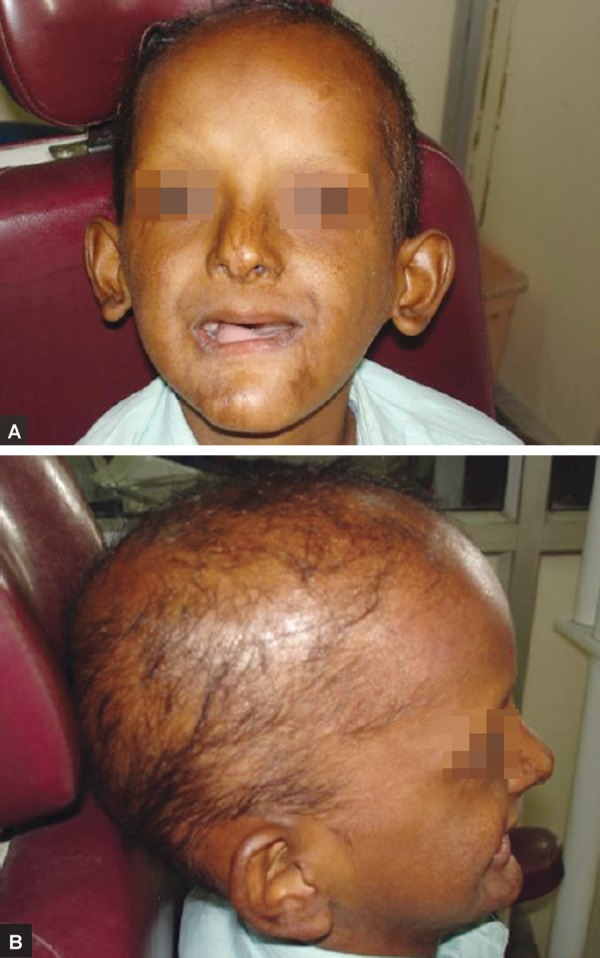
Pretreatment photographs

**Fig. 2:**
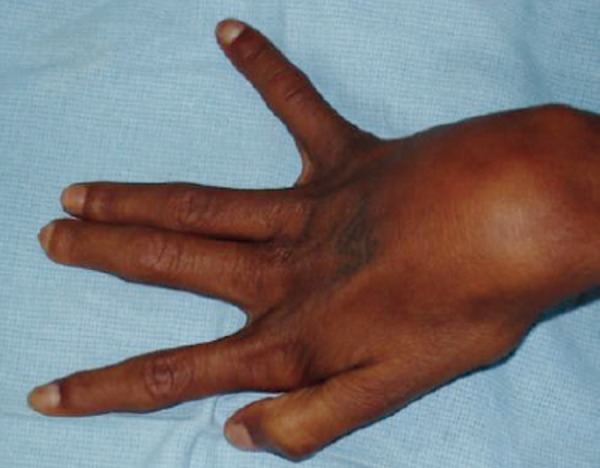
Onchodysplasia

The patient had typical characteristics (Figs 1A and B) of ectodermal dysplasia such as protuberant supraorbital ridge, saddle nose, frontal bossing, sparse hairs, scant eyelashes and eyebrow. The skin of the patient had maculopapular eruptions and he gave history of recurrent infections. He had onchodysplasia (Fig. 2).

**Fig. 3:**
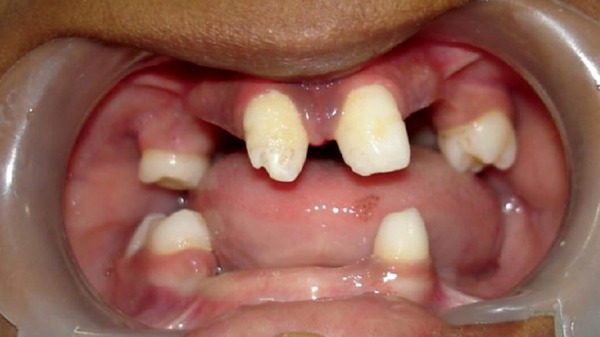
Intraoral view

Intraoral examinations (Fig. 3) revealed partial anodon-tia of both maxillary and mandibular arches. The patient showed normal salivation. In maxillary arch there were two permanent central incisors, which were conical shaped and hypo plastic and two right and left deciduous second molars. In mandibular arch there were second deciduous molar on right side and one permanent first molar on same side and one permanent cuspid on left side.

**Fig. 4:**
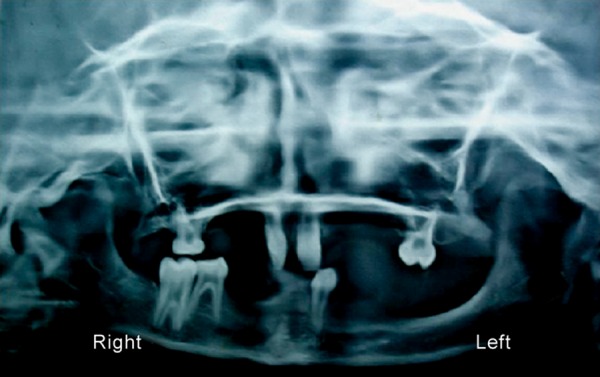
Preoperative orthopantograph

The alveolar ridges were underdeveloped. Radiographic examination (Fig. 4) revealed poor bony support of central incisors and all deciduous teeth. OPG revealed absence of other tooth buds.

After consultation with the pediatrician, oral surgeon, a detailed treatment planning for this patient was formulated which consisted of:

- Extraction of all deciduous teeth and maxillary permanent central incisors under antibiotic prophylaxis.
- Endodontic treatment of lower left cuspid and lower right first molar.
- Fabrication of upper complete denture against lower conventional overdenture.

**Fig. 5:**
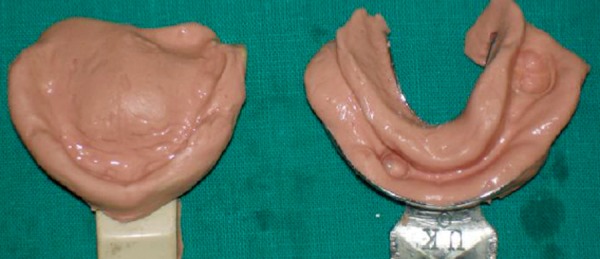
Primary impression with alginate

**Fig. 6:**
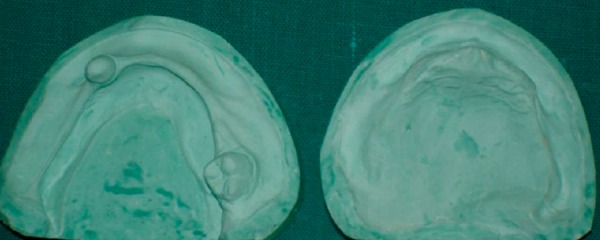
Diagnostic model after extraction

The objective was to preserve the health and restore function to meet the needs of developing stomatognathic system.

**Figs 7A and B:**
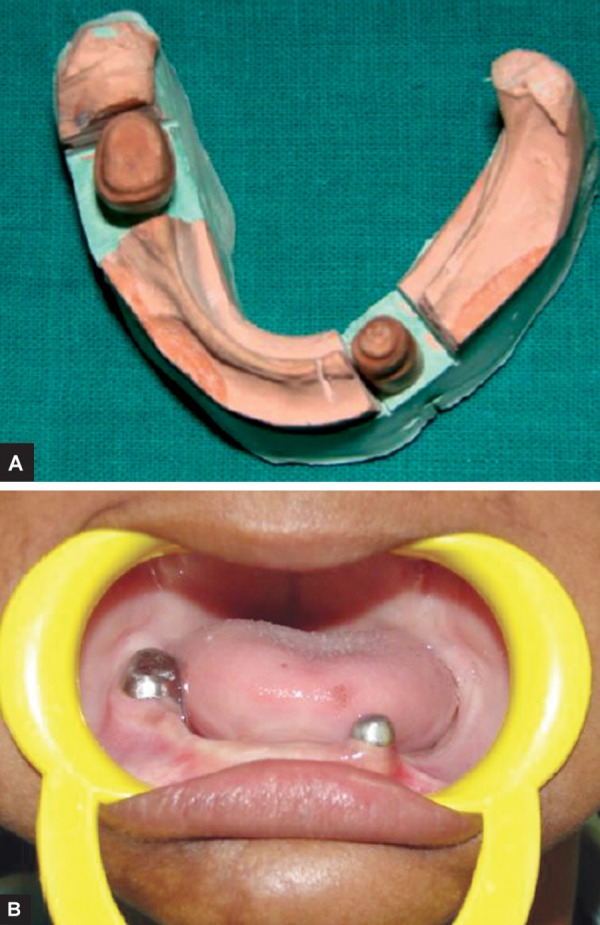
Metal coping on mandibular teeth

Then patient did not report to the department for 2 to 3 months because he was suffering from Chickengunya. After that he had completely lost his voice and had tumbling gait due to lack of neuromuscular coordination. His oral examination revealed paralyzed right side of tongue because on protrusion tongue deviate to left side. He showed uncontrolled mandibular movements with increased salivation.

Maxillary and mandibular primary impressions were made by irreversible hydrocolloid diagnostic impressions (Fig. 5) were made in irreversible hydrocolloid (alginoplast) material instead of impression compound and the diagnostic casts were made (Fig. 6).

We evaluated the coronal structure of the existing teeth, axial wall inclination and relative position of teeth to determine the path of insertion, retention and stabilization of metal coping for fabrication of mandibular overdenture.

An impression was taken with an individual tray using addition silicon rubber base material (Provil Novo Heraeus Kulzer GmbH). The metal copings (Figs 7A and B) were cast with a Cr-Ni based metal alloy. The copings were cemented to the teeth using polycarboxylate cement (Sankin Carlon SMFP Tokyo, Japan).

**Figs 8A and B:**
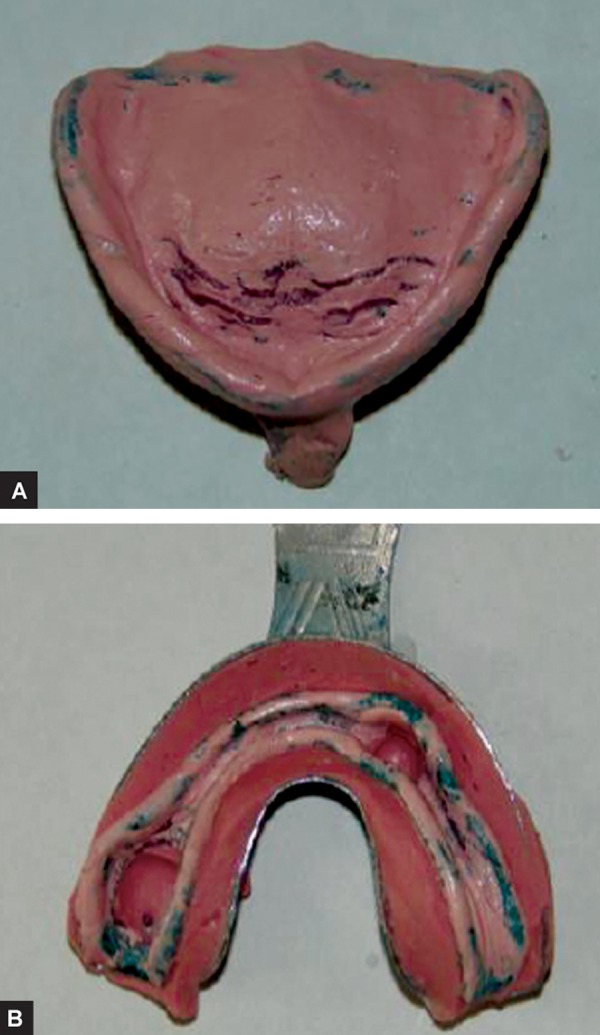
Final impressions

Final impressions (Figs 8A and B) were made with zinc oxide eugenol impression paste (DPI impression paste).

**Fig. 9:**
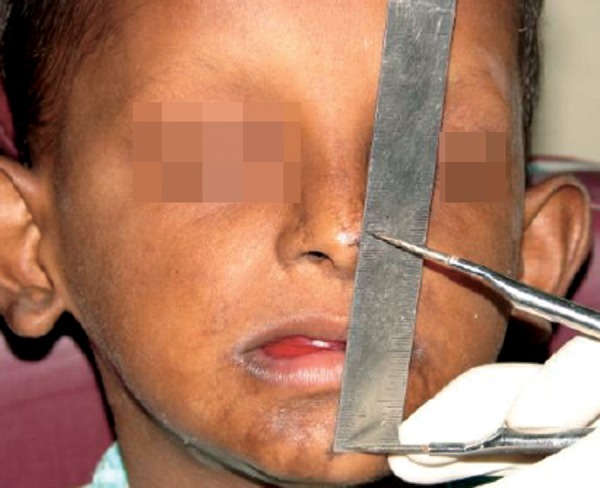
Jaw relation record

**Fig. 10:**
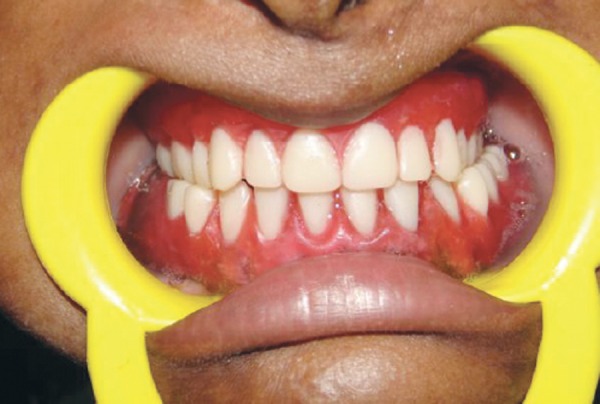
Teeth arrangement, and try-in

**Figs 11A and B:**
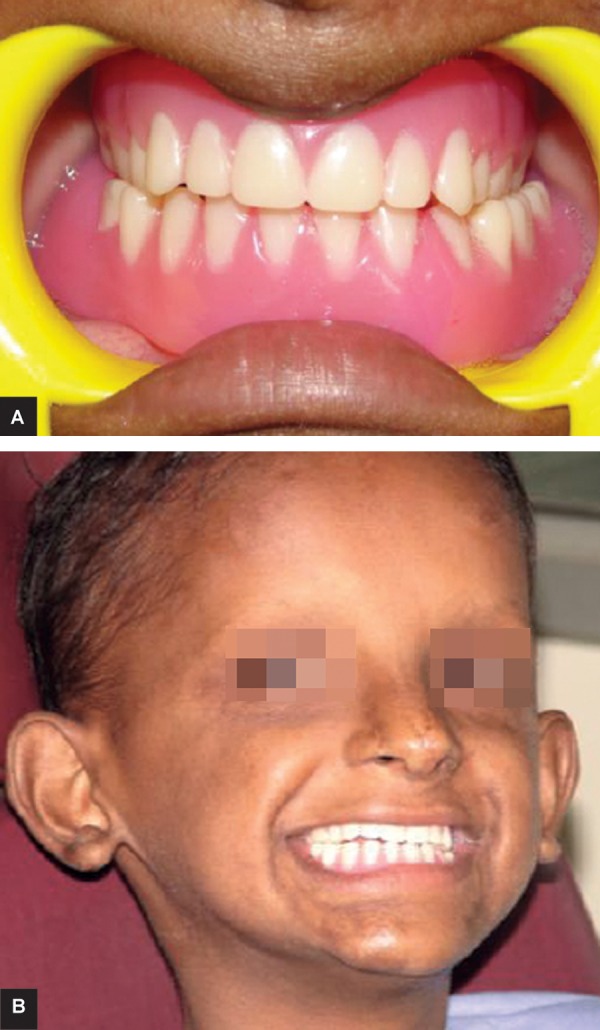
Post-treatment photographs

After jaw relation recording (Fig. 9) teeth were arranged as par the jaw relationship using 20 degree acrylic teeth (Fig. 10). The complete maxillary denture (Figs 11A and B) and conventional mandibular over denture were prepared using heat polymerizing acrylic resin.

## DISCUSSION

Complete dentures, partial dentures or overdentures are often part of treatment in patients with HED. Whenever teeth are present for support, overdenture are a desirable treatment option. One of the important advantages of overdenture is preservation of alveolar bone.^9,10^

In recent years, endosseous dental implants have been recognized as an important alternative for ED patients to support, stabilize, and retain the prosthesis. Considering the age and potential growth of our patient, it was deemed better to postpone osseointegrated implants.

Prosthodontic management of this patient was challenging for all steps right from impression making to use of dentures because of following problems:

- Fragile mucosa
- Increased salivation
- Poor alveolar ridge
- Underdeveloped maxilla
- Paralyzed right side of tongue
- Poor neuromuscular control and uncontrolled mandibular movements.

Maxillary and mandibular primary impressions were made by irreversible hydrocolloid impression (alginoplast) material instead of impression compound because of presence of fragile mucosa.

The slopes of the axial walls of teeth were made nearly parallel to enhance retention and stabilization of overdenture.

Final impressions were made with zinc oxide eugenol impression paste (DPI Impression paste).

During recording of jaw relationship, vertical dimension of occlusion was reduced slightly to reduce vertical forces on underdeveloped alveolar ridge. We planned cross arch arrangement of teeth because maxillary arch was collapsed and mandibular arch was wider posteriorly. Bilateral balanced occlusion was done using 20° acrylic teeth to reduce lateral forces on the residual alveolar ridges. The complete maxillary denture (Fig. 10) and conventional mandibular over denture were prepared using heat polymerizing acrylic resin.

## SUMMARY AND CONCLUSION

Child with HED often associated with dental problems and thus they suffer not only from functional difficulties but also poor esthetics. Early treatment is essential to encourage a normal physiological development and to improve function of stomatognathic system as well as to enhance esthetic to control psychological disturbances. The treatment part often involves team approach of different specialists such as oral and maxillofacial surgeons, pedodontist and prosthodontists, etc. Complete maxillary dentures and conventional mandibu-lar overdentures as a part of treatment in patients with HED could provide good functional, physiological and esthetic results. Whenever teeth are present for support, overdenture are a desirable treatment option.
